# Interval estimation of thermal summation parameters in forensically important insects

**DOI:** 10.1038/s41598-025-19926-3

**Published:** 2025-10-15

**Authors:** Jędrzej Wydra, Łukasz Smaga, Szymon Matuszewski

**Affiliations:** 1 Laboratory of Criminalistics, Adam Mickiewicz University, al. Niepodległości 53, 61-714 Poznań, Poland; 2 Center for Advanced Technologies, Adam Mickiewicz University, ul. Uniwersytetu Poznańskiego 10, 61-614 Poznań, Poland; 3 Department of Mathematical Statistics and Data Analysis, Adam Mickiewicz University, ul. Uniwersytetu Poznańskiego 4, 61-614 Poznań, Poland

**Keywords:** Forensic entomology, PMI estimation, Development constants, EM algorithm, Machine learning, Computational biology and bioinformatics, Computational models, Machine learning, Probabilistic data networks, Applied mathematics, Statistics

## Abstract

Estimating time of death based on entomological evidence commonly relies on the “law of total effective temperature”, which requires developmental parameters of specific insect taxa. These are often calculated using the method of Ikemoto and Takai. However, this approach has key limitations. Most importantly, the lack of interval estimates may give the false impression of population homogeneity, which contradicts the substantial variation typically observed in insect populations. In this study, we propose an alternative method. It estimates interval values for developmental parameters while simultaneously identifying component populations within a dataset. The method involves fitting a finite mixture of Weibull distributions to development time data using the Expectation-Maximization (EM) algorithm. This allows for the inclusion of individual-level variability in the estimation process. We tested the method using previously published developmental data on two beetle species, *Creophilus maxillosus* and *Necrodes littoralis* (Staphylinidae). Our approach yielded 95% intervals with coverage close to the nominal level, in contrast to Ikemoto and Takai’s method, which captured only 59% and 75% of actual cases, respectively. These findings suggest that our method improves the accuracy of insect-based postmortem interval estimates in forensic entomology and, more broadly, provides a general framework for interval estimation of developmental parameters applicable in thermal ecology and applied entomology.

## Introduction

The main application of forensic entomology is estimating the time of death based on the presence of insects, their parts, or residues of their activity on corpses^1,2^. From an entomological perspective, the postmortem interval (PMI) is divided into several subintervals depending on the evidence or the method used for its analysis. In the case of the method based on developmental models, which is the focus of this article, the pre-appearance interval (PAI) and the development interval (DI) are usually distinguished^3^. In the case of empty puparia, additionally the post-eclosion interval (PEI) is also used^4,5^.

PAI refers to the time elapsed from death until the first appearance of a given insect taxon on the corpse. Its length is difficult to estimate, and only a few methods, datasets, or models have been developed for this purpose^3^. Due to these difficulties, PAI is sometimes omitted. On the other hand, some authors attempt to estimate PAI and include it in the final conclusions regarding PMI^6^.

PEI denotes the time elapsed from when a fly eclosed (left puparium) until the discovery of the corpse and related empty puparium. Currently, there are no practically useful methods for estimating this interval. Therefore, the optimal solution is to assume that the length of PEI is zero and to conclude on the minimum PMI based on estimates of insect age and PAI used^5^.

DI represents the time during which an insect developed. Given the above assumptions, PMI in some cases can be reasonably reduced to DI. To differentiate, this is usually referred to as the minimum PMI (PMI_min_)^7^. The insect development is very predictable and closely linked to the temperature in which insects have been developing^1,8,9^. Consequently, the length of the DI is relatively easy to estimate, provided that there are high-quality temperature data from the death scene and robust reference development data for insects used in the estimation^1,9^. Reference development data are usually collected through the laboratory rearing of insects under several constant temperatures. By observing insect development, researchers can determine the time required to reach particular developmental landmarks at a given temperature. With this information, a so-called developmental model can be derived. This typically involves fitting some regression model to the development data.

There are numerous models to describe the relationship between the length of DI and temperature. One common approach is to express this relationship in terms of the development rate, which is the arithmetic inverse of development time (the length of DI). In this case, the dependent variable is the development rate, while the independent variable is the temperature^9–16^.

Insects effectively develop within a range defined by two biologically important temperatures: the developmental threshold and the upper lethal temperature. Below the developmental threshold, it is too cold and insect development pauses; above the lethal temperature, it is too hot and insects die^9^.

The common practice is to describe the above relationship using an exponential function. However, fitting such a model is computationally demanding, and specific conclusions about development parameters are highly dependent on the specific model chosen^16^.

In forensic entomology, it is not necessary to study the development rate in details, so its linear approximation is usually sufficient^9,11,17^. This simplifies both the model fitting and most importantly, its subsequent application in casework. Thus, the basis for consideration is the so-called law of total effective temperature^11^, expressed by the formula:

1 $$\begin{aligned} k = D(T - t_0) \end{aligned}$$

where *k* is the developmental constant, $t_0$ is the developmental threshold, *D* is the length of the DI, and *T* is the temperature at which insect development occurred. Here, *k* and $t_0$ are treated as parameters. Linear degree-day model^17^ was effective and mathematically most obvious way to model development rate:

2 $$\begin{aligned} \left( \frac{1}{D} \right) (T) = \frac{T}{k} - \frac{t_0}{k} \end{aligned}$$

By applying linear regression and treating the temperature as an independent variable, the values of the parameters *k* and $t_0$ could be easily found. Here, $\frac{1}{k}$ and $\frac{t_0}{k}$ are treated as parameters.

Subsequently, Ikemoto and Takai^11^ identified three problems with this approach: 1) the linear degree-day model does not allow for the identification of optimal temperature ranges, 2) it assumes a constant variance of the variable $\frac{1}{D}$ across all temperatures, and 3) it neglects the random error of the temperature variable *T*. Considering these issues, Ikemoto and Takai^11^ proposed their linearization:

3 $$\begin{aligned} DT(D) = t_0D + k \end{aligned}$$

which remains a very popular technique for determining developmental parameters in applied entomology, especially in forensic entomology. In the above formula, terms $t_0$ and *k* are treated as parameters.

Years of using the Ikemoto and Takai’s method have shown that it also suffers from certain issues. For instance, it is often applied to a limited number of data points, typically by reducing observations from each temperature to a single value (e.g., the median), resulting in approximately 6–13 points for regression (Table 1). This can be seen as an unnecessary reduction of information. Although the method can obviously be applied to full datasets (with many data points), the original paper tested the method on aggregated values^11^, which likely contributed to the widespread practice of applying it in this way. In addition, Ikemoto and Takai themselves suggested combining their linearization with Reduced Major Axis (RMA) regression, but this may cause the estimated parameter values to depend on the experimental design (for further details, see section *Mathematical Properties of Ikemoto and Takai’s Method* of Supplementary Materials).

Another issue with the Ikemoto and Takai’s method is that it assumes data analysis in the form of *DT* vs. *D*, which means that the variable *D* is present on both axes. These limitations lead to poor mathematical properties, which, in specific cases, may render the model less reliable. Specifically, this violates the assumptions of probabilistic models relying on the Central Limit Theorem. As a consequence, the values of the standard errors may lack a clear probabilistic or biological interpretation (see section *Mathematical Properties of Ikemoto and Takai’s Method* and Figure S1 of Supplementary Materials for further details).

**Table 1:** Number of data points used to fit Ikemoto and Takai’s model in selected studies on the temperature-dependent development of forensically useful insects. Counts were obtained before excluding data points according to Ikemoto and Takai’s method.

Order	Family	Species	No. of data points	Reference
Coleoptera	Cleridae	*Necrobia rufipes*	6	^18^
	Dermestidae	*Dermestes tessellatocollis*	7	^19^
	Nitidulidae	*Nitidula rufipes*	7	^20^
		*Omosita colon*	7	^21^
	Staphylinidae	*Creophilus maxillosus*	10	^22^
		*Necrodes littoralis*	10	^23^
		*Thanatophilus micans*	10	^24^
		*Thanatophilus sinuatus*	7	^25^
Diptera	Calliphoridae	*Chrysomya albiceps*	13	^26^
		*Chrysomya albiceps*	11	^27^
		*Chrysomya megacephala*	7	^28^
		*Chrysomya pinguis*	7	^29^
		*Chrysomya rufifacies*	7	^30^
		*Lucilia cuprina*	6	^31^
	Muscidae	*Fannia canicularis*	9	^32^
		*Muscina stabulans*	7	^33^
	Phoridae	*Megaselia scalaris*	7	^34^
	Sarcophagidae	*Boettcherisca peregrina*	7	^35^
		*Sarcophaga dux*	7	^36^

Moreover, there is a debate as to whether a point estimate of the *k* parameter is biologically appropriate. It turns out that factors such as food quality or air humidity influence the value of the *k* parameter^37^. Furthermore, recent studies have demonstrated a correlation between the value of *k* for individual insects and their size, expressed through body weight or length^25,38,39^. This implies that methods such as those proposed by Ikemoto and Takai or Campbell and colleagues allow for the calculation of only an average *k* value for the population, without accounting for its intra-population variability. As a result, the obtained estimate of *k* from the model is accurate only in an average sense (i.e., considering multiple cases, the mean estimation error should be close to zero).

A development time of an insect estimated in a specific case based on the average *k* value may be subject to significant error. From the perspective of forensic practice, such limitations of point estimation are particularly relevant. Point values may appear overly precise to legal practitioners such as judges or juries, who typically lack biological or statistical training and may not appreciate the uncertainty underlying such estimates^40–42^. This creates the risk of misinterpretation in a courtroom setting, a problem acknowledged in the forensic science literature^42,43^. Interval estimates, in contrast, explicitly represent the variability of insect development and thus provide a more transparent tool in forensic casework.

Beyond forensic applications, interval estimation could also be of broader interest in thermal ecology and applied entomology, as it captures intra- and interspecific variability and can serve as a basis for comparative analyses across insect taxa^37–39^.

The literature identifies two solutions to the problem of point estimates of parameter *k*. First, the *k* values can be calibrated retrospectively by accounting for insect size and sex using the linear approximation^38,39,44^. As a result, a parameter value ‘tailored’ to a specific individual can be obtained. The retrospective calibration of *k* can be performed without empirical data as proposed by Wydra et al.^44^ or by using a linear model derived using the data as proposed by Matuszewski & Frątczak-Łagiewska^38^. However, this approach has so far been validated for only a limited number of species. Moreover, it should be applied with caution, especially since later studies have shown that it is ineffective for certain species, such as *Thanatophilus sinuatus*^25^.

Second, an alternative to point estimation is interval estimation. Defining a range for the *k* parameter would cover multiple potential cases, which could be particularly useful in the estimation of insect age and PMI, as it would establish a range of possibilities that could later be narrowed down using other evidence. The literature has paid little attention to interval estimation of the *k* parameter. Ikemoto and Takai^11^ addressed this issue, but not in a fully satisfactory way, as they relied on the standard error. Such an approach constructs an interval for potential average values rather than for potential actual values. By definition, the intervals derived using standard errors will be too narrow.

In forensic entomology there were also attempts to estimate PMI in the form of an interval^6^. It turned out that intervals with high coverage of actual cases tend to be very wide, which may limit their practical applicability. Therefore, Matuszewski and Mądra-Bielewicz^45^ suggested controlling the interval width and use the narrower intervals for investigative purposes and wider intervals for evidentiary purposes.

The aim of this study is to propose a method for interval estimation of the parameter *k* for an insect population in a way that accounts for individual insect characteristics influencing its value while allowing for manual adjustment of the interval width. Furthermore, given concerns about the practical utility of wide intervals, the method should incorporate as much data as possible to achieve the highest possible precision. At the same time, ensuring strong mathematical properties of the model should significantly contribute to maintaining its reliability and adaptability to different cases.

In the next sections, we present the proposed method in detail (technical aspects are discussed in the Supplementary Materials) and demonstrate its application for two beetle species of forensic importance (*Creophilus maxillosus* and *Necrodes littoralis*). We present the results of model fitting, the identification of component populations, and the simulation of insect age estimation. We also compare the empirical performance of our method with the classical approach of Ikemoto and Takai, particularly in terms of coverage probability. Finally, we discuss the biological and forensic implications of our findings and evaluate the method’s strengths and limitations.

## Results

### *Creophilus maxillosus*

#### Coefficients of the fitted model

**Fig. 1:**
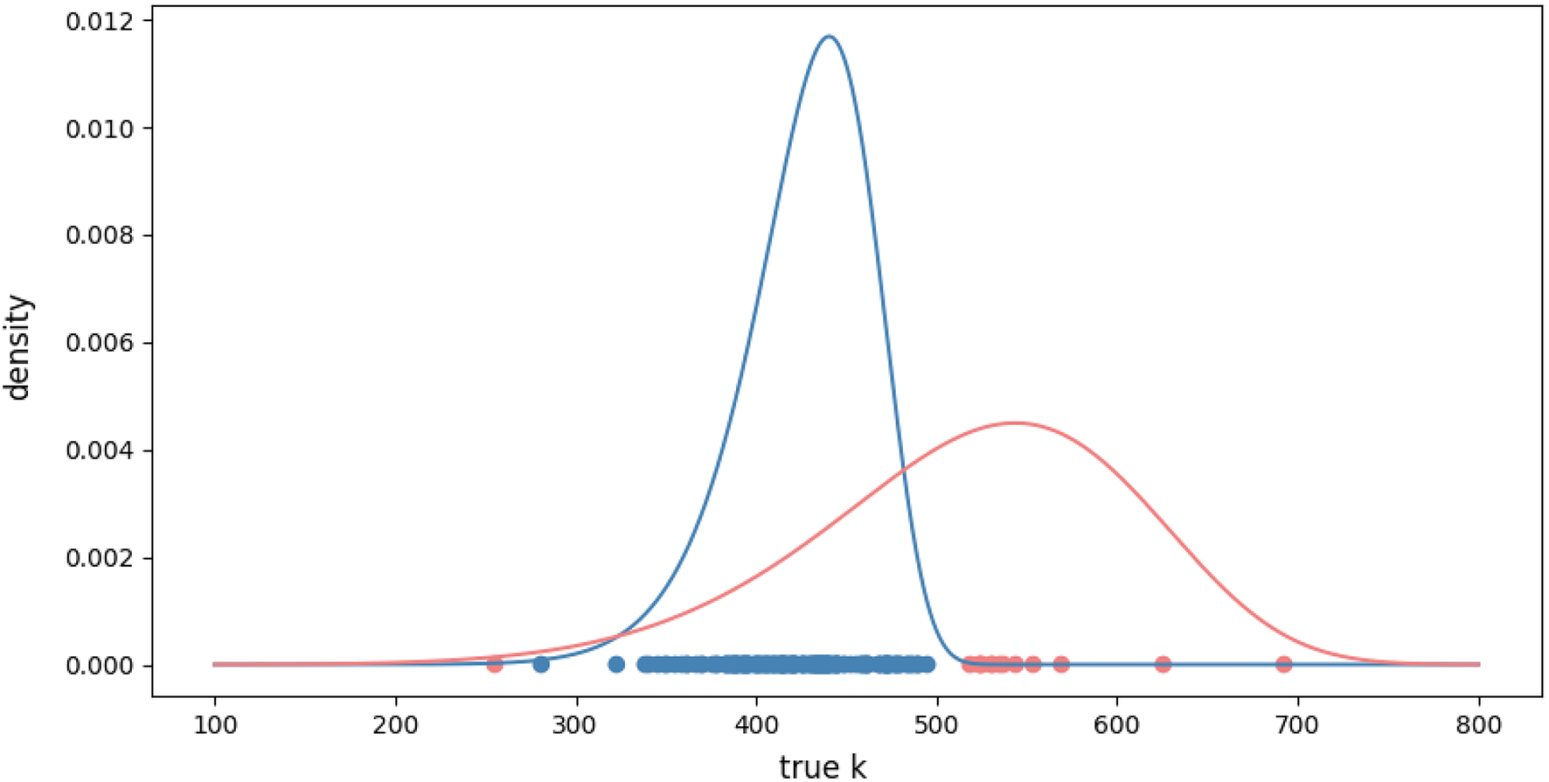
Density functions for the detected populations in the *Creophilus maxillosus* dataset. The regular experiment results are marked in blue, and outliers are marked in red.

For *Creophilus maxillosus*, our algorithm distinguished two component populations, fitting each to a Weibull distribution. The coefficients of the fitted distribution are presented in Table 2. The separation of these populations is illustrated in Fig. 1.

**Table 2:** Coefficients of the mixture model of Weibull’s distribution for the *Creophilus maxillosus* dataset.

Parameter	Large-insect population (blue)	Small-insect population (red)
$w_l$	0.87	0.13
$\kappa _l$	14.03	6.72
$\lambda _l$	442.74	556.66
$t_0^{(l)}$	11.25	11.15

#### Component populations

The red population contained far fewer individuals than the blue population. Approximately seven times fewer (see the $w_l$ coefficients in Table 2). According to the U-test, there were no significant differences in the insect size between the two populations (Table 3, *p*-value $> 0.7$) and the chi-squared test revealed no significant differences in the proportion of males and females between populations (*p*-value $> 0.5$). Additionally, the red population exhibits extreme or outlier values of *k* and DI (Fig. 1). Therefore, we consider the blue population to represent the regular experimental results, while the red population is treated as outlier observations.

The standard deviation of random variable *K* is about 30% smaller in the blue (regular) population compared to the combined population.

**Table 3:** Mean insect length in each population of *Creophilus maxillosus*.

Population	Length [mm]
Regular-insect population (blue)	19.57
Outlier population (red)	19.21

#### Insect age estimation

When estimating the insect age using the proposed method, the empirical coverage probability was 0.94 after excluding outliers and 0.97 when calculated using the full dataset, both values being close to the expected level of 0.95. In contrast, the interval derived from the approximation based on Ikemoto and Takai’s method yielded a coverage probability of only 0.59 when applied to the entire dataset (Table 6).

### *Necrodes littoralis*

#### Coefficients of the fitted model

**Fig. 2:**
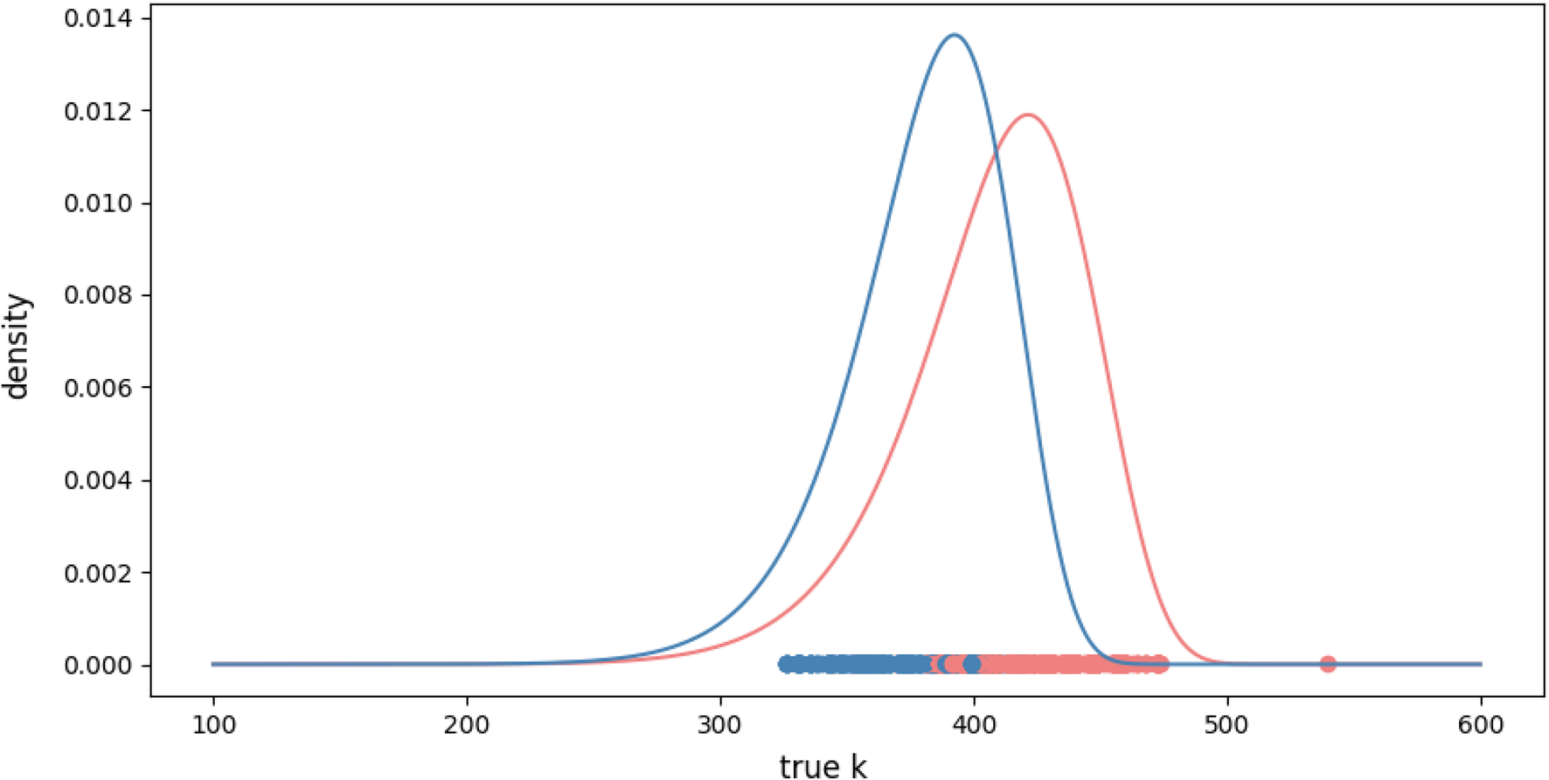
Density functions for detected populations in the *Necrodes littoralis*’ dataset. The large individuals are marked in blue, and the small individuals are marked in red.

For *Necrodes littoralis*, our algorithm distinguished two component populations, fitting each to a Weibull distribution. The coefficients of the fitted distribution are presented in Table 4. The separation of these populations is illustrated in Fig. 2.

#### Component populations

The sizes of the red and blue populations are similar (see $w_l$ coefficient in Table 4). Additionally, the red population shows a systematically longer time of development (Fig. 2), and according to the U-test (*p*-value $< 10^{-10}$ ) individuals in this population are significantly smaller than those in the blue population (Table 5), but there are not any significant differences in proportion of males and females between populations according to chi-squared test (*p*-value  $> 0.3$). We described the blue population as the large-insect population and the red population as the small-insect population.

The standard deviations of random variable *K* in the large-insect population (blue) and small-insect populations (red) are very similar and about 30% smaller than in the combined population.

#### Insect age estimation

When estimating the insect age using the proposed method, the empirical coverage probability was 0.97, which is close to the expected level of 0.95. In contrast, the interval based on the approximation derived from Ikemoto and Takai’s method yielded a coverage probability of only 0.75 (Table 6).

**Table 4:** Coefficients of the Weibull mixture model for the *Necrodes littoralis* dataset.

Parameter	Large-insect population (blue)	Small-insect population (red)
$w_l$	0.41	0.59
$\kappa _l$	14.57	13.67
$\lambda _l$	394.44	423.95
$t_0^{(l)}$	9.52	10.21

**Table 5:** Mean insect length in each population of *Necrodes littoralis*.

Population	Length [mm]
Large-insect population (blue)	17.75
Small-insect population (red)	16.96

### Additional results

The additional results for *Necrodes littoralis* and *Creophilus maxillosus* are provided in the Supplementary Materials. Figures S2–S3 show the relationship between development time and temperature. Tables S1–S8 present the detailed performance of the proposed method across different temperatures. The Supplementary Materials also include a practical application example based on the *Necrodes littoralis* data. Tables S9–S15 provide the values of the parameters *k* and $t_0$ for interval estimation at different expected coverage probabilities for both species.

**Table 6:** Comparison of interval estimates and empirical coverage probabilities for the proposed and classical methods across species and conditions.

Species / Condition	Proposed method		Ikemoto and Takai’s method	
	Interval (k)	Coverage probability	Interval (*k*)	Coverage probability
*Necrodes littoralis*	[315, 461]	0.97	[421, 517]	0.75
*Creophilus maxillosus* (all)	[339, 600]	0.97	[379, 456]	0.59
*Creophilus maxillosus* (w/o outliers)	[341, 486]	0.94	–	–

## Discussion

The concept of component insect populations has been widely discussed in the literature, particularly regarding the effects of various environmental factors such as food quality and humidity on the variability of developmental parameters. This variability challenges the notion of true constancy in developmental parameters within a single species or population, suggesting the presence of distinct groups with differing developmental characteristics^37–39,46–49^. These groups, referred to as component populations in this study, can be identified using our algorithm without the need for detailed data on external variables like food quality or humidity. This method facilitates the subsequent differentiation of populations based on experimental contexts, offering a practical advantage over approaches requiring extensive datasets on environmental conditions. By focusing on inherent patterns within the data, our algorithm supports a streamlined and adaptive process for detecting developmental differences across the populations identified by the method within the experimental sample.

Recent findings indicate that developmental parameters correlate with insect size. Matuszewski and Frątczak-Łagiewska^38^ and Gruszka and Matuszewski^39^ reported a statistical relationship between insect size and the parameter *k*, modeled using linear regression. Subsequently, Wydra et al.^44^ proposed an approximation method for linear adjustment of developmental constants, accounting for insect size. In all of these studies, the developmental parameter was first estimated and only subsequently calibrated, rather than being calibrated during the derivation of the developmental model. This raises certain concerns, as it involves estimation based on estimation, which may increase the error of the initial estimate, leading to less precise results^50^. The current method addresses this issue by calculating developmental parameters while directly considering insect size. In both cases we examined, insects from the cluster with a longer development time were smaller than the others, although this difference was statistically significant only for *Necrodes littoralis*. Notably, it was also observed in *Creophilus maxillosus*. Importantly, calibrating the *k* value directly within the current method remains feasible, as calibrating incorporates a much broader range of insect sizes than the identification of component populations. As a result, the value subjected to calibration would carry a smaller error, and consequently, the calibrated value itself would likely also be more accurate. Nevertheless, this approach requires further research.

A drawback of our method is that it is not entirely clear whether the clusters are determined truly by insect size. The EM algorithm detects a latent variable that is not explicitly defined; therefore, it does not determine what that variable represents^51,52^. Based on the results of our tests, insect size is associated with the observed differentiation of clusters, which is consistent with previous studies^25,38,39^. Furthermore, we expected that the relationship between development time and size for *Creophilus maxillosus* would be approximately half as strong as for *Necrodes littoralis*^44^, and this prediction was confirmed by the results. The algorithm identified two distinct populations differing in size for *Necrodes littoralis*, whereas for *Creophilus maxillosus*, it likely identified just outliers. Nevertheless, we had relatively few data points in this case (approximately 170 observations), and with a larger sample size, the algorithm would probably separate the populations more effectively.

The hypothesis that insect size drives cluster differentiation remains tentative and requires further research. For example, in *Thanatophilus sinuatus*, only a weak correlation between size and *k* parameter was observed, with limited practical relevance^25^, yet it remains detectable. Nevertheless, with a sufficiently large sample, the algorithm would likely detect more than two component populations, as suggested by the findings of Matuszewski and Frątczak-Łagiewska^38^ and Gruszka and Matuszewski^39^. By a sufficiently large sample, we should understand a size exceeding that used for *Necrodes littoralis* (approximately 1,000 observations), likely requiring over 2,000 observations. However, factors other than insect size may also contribute to cluster differentiation, such as genetic variation or biochemical markers.

At this stage, for *Creophilus maxillosus* we suggest using the developmental parameters of the regular-insect population in all cases. For *Necrodes littoralis* we tentatively recommend applying the parameters of the large-insect population for clearly large individuals and those of the small-insect population for clearly small individuals. According to our data, and by convention, large individuals are those exceeding 17.34 mm; however, this threshold may be specific to our laboratory population. Therefore, it should be applied with caution. In practice, it seems preferable to rely on the judgment of experts: if an individual is evaluated as large, the parameters for the large-insect population should be used, and analogously for small individuals.

Most importantly, our method allows for interval estimation of insect development time, while providing control over the width of the interval, ensuring that the estimation includes the desired percentage of actual cases (Tables S11-S15 in the Supplementary Materials). We present 95% intervals; however, obtaining intervals with other coverage levels is possible by selecting the appropriate quantiles of the Weibull distribution using the parameters provided in Table 2 or Table 4 (examples are provided in the Supplementary Materials). This explicitly ties the interval width to a chosen probability level and aligns with recommendations to report probability-bearing PMI statements and uncertainty in forensic entomology^53^. The interval estimation is particularly significant as it accounts for the natural variability in development time and encompasses many real-world cases^6^. In contrast, point estimation only represents a model average case. A direct comparison with the Ikemoto and Takai method further demonstrated that our approach provides interpretable interval estimates, whereas the classical method is limited to point estimation or ad hoc approximations.

It is not possible to determine the exact development time of insects in a specific case due to a random or natural biological variability and measurement limitations. Therefore, the estimation should account for a reasonable range of possible scenarios, providing a time interval during which the insects may have colonized the remains. This view is supported by simulation-based analyses of succession data, which quantify the inherent statistical uncertainty in PMI inference and motivate interval-based reporting^54^. When combined with PAI and the recorded temperature history, such colonization-time intervals can be propagated into probability-bearing PMI intervals (e.g., 90% or 95%), offering a more transparent alternative to point estimates.

This approach, however, has an inherent weakness: interval estimates are inevitably wide, and excessively wide intervals may lose practical utility. One potential solution, as suggested by Matuszewski and Mądra-Bielewicz^45^, involves using estimation intervals narrower than those covering 95% of cases. This approach increases the practical usefulness of the estimates but reduces their accuracy. Another possible method is to combine information from multiple pieces of evidence^43,55,56^. Recently, it has been demonstrated that when multiple pieces of insect evidence are used, coverages of PMI intervals are generally much higher than when single pieces of evidence are applied^45^.

The proposed approach offers improved mathematical properties compared to previous solutions. In the procedure developed by Ikemoto and Takai, development time appears on both axes, meaning it is treated simultaneously as a random and a non-random variable^57,58^. This is problematic because development time should be considered a random variable (see section *Mathematical Properties of Ikemoto and Takai’s Method* of Supplementary Materials for further details).

Moreover, this inconsistency affects the reliability of their estimates, as illustrated in Table 6. The intervals produced by our method are substantially wider than those obtained using Ikemoto and Takai’s model. Although both techniques aim to cover 95% of actual cases, the coverage probability in the case of Ikemoto and Takai’s procedure is notably lower; covering only about 59% of observations for *Creophilus maxillosus* and about 75% for *Necrodes littoralis*. In contrast, with our approach, the coverage probability remains close to the intended 95% in both cases.

The most significant drawback of the proposed method is likely its inaccuracy in the separation of component populations. The algorithm divides data into populations based on probability. As a result, it may make mistakes, and cases may be assigned to wrong populations. However, such misclassifications are probably rare and from a statistical point of view, it is sufficient that the vast majority of observations are correctly classified^59^.

An additional limitation concerns the application of our approach to dipteran datasets. In studies on flies, only the fastest-developing individuals are used to represent the group^60,61^. While this practice reduces the potential for analyzing intrapopulation variability, our method can still be applied to such data, yielding interval estimates for the fastest-developing fraction of the population. Nevertheless, the full capacity of the method to identify component populations and account for developmental variability can only be realized when datasets include the entire range of developmental times, as is the case in our beetle examples.

The last weakness of the method is its lack of integration with popular data analysis software. To address this, the first author has provided Python scripts on his GitHub repository as well as a ready-to-use software prototype in a Jupyter Notebook, available via Google Colab. Both resources will be updated and maintained regularly.

In summary, the proposed method offers a significant advancement in forensic entomology by enabling the detection of component insect populations without requiring detailed information on environmental factors, e.g. food quality. It also provides accurate and controllable interval estimates of insect thermal summation parameters, addressing key weaknesses of earlier methods, including logical inconsistencies and unrealistic confidence intervals. This focus on interval estimation aligns with prior recommendations in forensic entomology, which emphasize probability-based reporting and provide formal statistical procedures for constructing confidence sets for insect development time based on developmental stage^62^. The proposed method performs favorably compared to bootstrap-based estimators, and its capacity to directly incorporate insect size into the estimation process enhances both its precision and interpretability. Although the algorithm may occasionally misclassify individual cases, its statistical robustness ensures that the vast majority of observations are correctly assigned. Finally, the ability to adjust the width of the estimation intervals makes this approach both flexible and practical for diverse forensic applications. Overall, the method represents a reliable and versatile tool for improving the accuracy of the thermal summation parameters used in postmortem interval estimation.

Nevertheless, the present findings should be validated using data from populations beyond those studied here. In particular, testing the method across geographically diverse regions (e.g., Southern and Northern Europe, or tropical areas) would provide evidence for its robustness and generalizability. From a mathematical perspective, the procedure appears promising and should in principle be applicable wherever earlier approaches such as those of Ikemoto and Takai or Campbell and colleagues have been used. Such external validation will therefore be essential to fully establish the method as a reliable tool in forensic casework worldwide.

## Methods

### Data

We utilized data about the temperature-dependent development of *Creophilus maxillosus* (174 observations) and *Necrodes littoralis* (954 observations) from previous studies supervised by one of the authors^22,23^. In both cases, the datasets consisted of five variables: length of DI, development temperature, insect sex, length, and weight. The data were collected during laboratory experiments aimed at describing insect development in various constant temperature conditions for forensic entomology purposes. The insects were reared in temperature chambers. The *Creophilus maxillosus* was studied at ten temperatures (10, 12.5, 15, 17.5, 20, 22.5, 25, 27.5, 30 and $32.5^\circ$ C), however mortality at the extreme temperatures (10, 12.5, and $32.5^\circ$ C) was very high, and no insects reached the adult stage. Therefore, only the remaining seven temperatures were used in the current analyses. The *Necrodes littoralis* was studied also at ten temperatures (14, 15, 16, 17, 18, 19, 20, 22, 26, and $30^\circ$ C). All insects were used in the analyses. For detailed descriptions of data collection methods, refer to the original papers^22,23^.

### Machine learning and data analysis

In this section we present a general idea of the study. Technical details are available in the Supplementary Materials. The full implementation, including data preprocessing and visualization, is available in the GitHub repository .

We assumed that *D* and *T* are random variables and that the results of the developmental experiment may not necessarily come from a single population, e.g. in Table 7, rows 1, 2, 4, and 6 may come from a different population than rows 3 and 5. Populations could be, for instance, regular experimental results and outliers, or large individuals and small individuals, or females and males. All observations are referred to as the combined population, while the distinct populations are called component populations.

**Table 7:** Selected data from the *Creophilus maxillosus* development experiment. Columns show the value of the development index *D* and the corresponding temperature *T* in °C.

No.	Development index *D*	Temperature [°C]
1	109.79	15.0
2	141.13	15.0
3	66.25	15.0
4	87.12	17.5
5	62.08	17.5
6	70.29	17.5

Then the EM algorithm and the finite mixture models (see Supplementary Materials) were used to detect the component populations within the combined populations of *Creophilus maxillosus* and *Necrodes littoralis* (analyzed separately) and estimated the value of $t_0$ for each species by treating it as a parameter of the probability distribution. Treating *D* and *T* as random variables, based on the formula $k = D(T - t_0)$, we have to assume that the developmental parameter *k* is also a random variable. Therefore, for clarity in notation, we will denote *K* as the random variable and *k* as its realization. Finally, using mixture models, we determined 95% confidence intervals for the estimator of the expected value of *K* and an interval estimate that captures 95% of the realizations *k* of *K*.

For clarity, the confidence interval is an interval that describes the uncertainty of estimating a population parameter; the narrower it is, the more precise the estimation is. On the other side, an interval estimate describes the variability of realizations of a random variable, i.e., how a given variable changes in real-life situations. The width of this interval reflects the variability of the random variable^63^ (for more details, see the Technical Details section in the Supplementary Materials).

A confidence interval should be used as a metric for the precision of estimation, while an interval estimate should be used as a tool to estimate real-life values. In the case of forensic entomology, interval estimate of realizations *k* can be used to estimate the length of DI as interval.

When detecting component populations, if one population was substantially larger than the others and there were no significant differences in individual size or in the proportion of males and females between populations, we considered it to represent regular experimental results, while the other populations were classified as outliers. We used the Mann-Whitney U-test to assess whether differences in individual size were significant, and the chi-squared test to evaluate differences in the proportion of males and females.

When the component populations were of similar size, we also applied the Mann-Whitney U-test to assess differences in individual size and the chi-squared test to assess differences in the proportion of males and females between populations. The population with significantly smaller individuals was classified as the small-insect population, while the population with significantly larger individuals was classified as the large-insect population.

To allow for a meaningful comparison between the newly proposed method and the classical approach of Ikemoto and Takai^11^, we evaluated their performance in estimating the insect age. Since the Ikemoto and Takai procedure provides only point estimates, we additionally considered a commonly observed but not recommended practice in which an interval is approximated as the mean estimate $\pm 1.96$ standard errors^64–66^. For each individual and each rearing temperature, we therefore constructed insect age intervals using our method according to its definition (detailed procedures are provided in the Supplementary Materials), and using the Ikemoto and Takai framework with the above approximation. The accuracy of both approaches was then assessed by calculating coverage probability, i.e. the proportion of observed values falling within the constructed intervals.

## Supplementary Information

Supplementary Information.

## Data Availability

The data analyzed in this study were obtained from previously published works and are not publicly available due to copyright and data sharing restrictions. Access to these data can be requested from the corresponding authors of the respective publications: 1. Frątczak-Łagiewska, K., Grzywacz, A. & Matuszewski, S. Development and validation of forensically useful growth models for central european population of Creophilus maxillosus l. (coleoptera: Staphylinidae). Int. J. Leg. Medicine 134, 1531–1545, (2020), https://doi.org/10.1007/s00414-020-02275-3 2. Gruszka, J. & Matuszewski, S. Temperature models of development for Necrodes littoralis l. (coleoptera: Silphidae), a carrion beetle of forensic importance in the palearctic region. Sci. Reports 12, 9689, (2022), DOI: https://doi.org/10.1038/s41598-022-13901-y The datasets were used with appropriate permissions solely for the purposes of this analysis. All additional data, materials, and code generated during this study are openly available in the GitHub repository (https://github.com/Jedrzej-Wydra/survival_analysis_em) and in supplementary materials.
