# Supplementary material for: Interval estimation of thermal summation parameters in forensically important insects
Source: Sci Rep. 2025 Oct 15;15:36038. doi: 10.1038/s41598-025-19926-3 (PMC12528742; doi:10.1038/s41598-025-19926-3)
Supplement: Supplementary file 1 — Supplementary Information. [file 41598_2025_19926_MOESM1_ESM.pdf]

Supplementary materials for “Interval estimation of thermal summation parameters in forensically important insects”

Jędrzej Wydra[1,2,*], **Łukasz Smaga**[3], **and Szymon Matuszewski**[1,2]

[1]Laboratory of Criminalistics, Adam Mickiewicz University, al. Niepodległości 53, Poznań 61-714, Poland
[2]Center for Advanced Technologies, Adam Mickiewicz University, ul. Uniwersytetu Poznańskiego 10, Poznań 61-614, Poland
[3]Department of Mathematical Statistics and Data Analysis, Adam Mickiewicz University, ul. Uniwersytetu Poznańskiego 4, Poznań 61-614, Poland
[*]corresponding author: jedrzej.wydra@amu.edu.pl

ABSTRACT

Estimating time of death based on entomological evidence commonly relies on the "law of total effective temperature", which requires developmental parameters of specific insect taxa. These are often calculated using the method of Ikemoto and Takai. However, this approach has key limitations. Most importantly, the lack of interval estimates may give the false impression of population homogeneity, which contradicts the substantial variation typically observed in insect populations.
In this study, we propose an alternative method. It estimates interval values for developmental parameters while simultaneously identifying component populations within a dataset. The method involves fitting a finite mixture of Weibull distributions to development time data using the Expectation-Maximization (EM) algorithm. This allows for the inclusion of individual-level variability in the estimation process.
We tested the method using previously published developmental data on two beetle species, *Creophilus maxillosus* and *Necrodes littoralis* (Staphylinidae). Our approach yielded 95% intervals with coverage close to the nominal level, in contrast to Ikemoto and Takai's method, which captured only 59% and 75% of actual cases, respectively. These findings suggest that our method improves the accuracy of insect-based postmortem interval estimates in forensic entomology and, more broadly, provides a general framework for interval estimation of developmental parameters applicable in thermal ecology and applied entomology.

Content of the Supplement

This Supplementary File contains additional content related to the article titled "Interval estimation of thermal summation parameters in forensically important insects". It includes: (1) Mathematical properties of Ikemoto and Takai's method, (2) Additional results for *Creophilus maxillosus*, (3) Additional results of *Necrodes littoralis*, (4) Example of practical application using *Necrodes littoralis* data and (5) technical details of the proposed method.

Mathematical properties of Ikemoto and Takai's method

Dependence of estimated coefficients on experimental design

According to Ikemoto and Takai's method, the parameters t_0 and k are obtained by fitting the linear model

$$DT = t_0 D + k$$

to data of the form DT vs. D, treating t_0 and k as parameters. Since both T and D are random variables, Ikemoto and Takai suggested using Reduced Major Axis (RMA) regression instead of the classical Ordinary Least Squares (OLS)[1].

However, this approach raises methodological concerns because the random character of D and T is different. The variable D represents development time and is directly observed in laboratory or field settings. The variable T, in contrast, is typically fixed by the researcher. Any randomness in T stems mainly from measurement error (e.g., slight deviations between the set and recorded temperature). Thus, the variance of T is largely controlled by experimental design. For example, given the set of temperatures $\mathbf{T_1} = (10, 14, 22, 25, 33, 35)$, the variance of T is 83.14, while for $\mathbf{T_2} = (18, 21, 22, 25, 26, 27)$ the variance is only 9.81.

In the RMA framework, parameter estimates depend on variances and covariances:

$$\hat{t}_0 = \text{sign}(S_{D,DT})\sqrt{\frac{S_{DT}}{S_D}}, \qquad \hat{k} = \overline{DT} - \hat{t}_0\,\overline{D},$$

where $S_{D,DT}$ is the covariance of DT and D; S_{DT} and S_D are their respective variances. Consequently, $\hat{t}_0$ and $\hat{k}$ may vary considerably depending on the chosen temperature set, which is an arbitrary decision of the researcher.

Consider a simple example. Assume the true parameter values are $k = 400$ and $t_0 = 8$. For each value of T, let D be normally distributed with mean $\mu = \frac{400}{T-8}$ and standard deviation linearly decreasing from 10 at $T = 10$ to 1 at $T = 35$. If the researcher rears 100 individuals at each temperature in $\mathbf{T_1}$ and applies Ikemoto and Takai's method to unaggregated data, the resulting estimates are $\hat{t}_0 = 8.12$ and $\hat{k} = 393.27$. For $\mathbf{T_2}$, the estimates become $\hat{t}_0 = 15.3$ and $\hat{k} = 195.4$. When aggregated data are used (e.g., median development time per temperature), the results are $\hat{t}_0 = 7.98$, $\hat{k} = 403.74$ for $\mathbf{T_1}$, and $\hat{t}_0 = 7.52$, $\hat{k} = 414$ for $\mathbf{T_2}$.

By comparison, the method proposed in this study yields $\hat{t}_0 = 8.11$, $\hat{k} = 406.72$ for $\mathbf{T_1}$ and $\hat{t}_0 = 8.43$, $\hat{k} = 409.93$ for $\mathbf{T_2}$. The above example is based on artificial data, so no direct recommendations regarding optimal experimental design can be drawn. However, it clearly demonstrates that the Ikemoto and Takai method is highly sensitive to the choice of experimental temperatures.

Statistical dependence between D and DT

The issue described above is related to the use of RMA regression. Even when reverting to OLS, an important structural problem remains. Specifically, the pair (D, DT) does not form an independent and identically distributed sample in the usual sense. In developmental experiments, T is fixed at discrete levels, and for each j such as $T = t_j$ the variable D has a conditional distribution $F_{D|T=t_j}$. Thus, the sample consists of random vectors with possibly different distributions,

$$\{(d_i, t_j d_i) : D \sim F_{D|T=t_j}, T = t_j\}$$

for $i = 1, ..., n$, $j = 1, ..., m$ and $m < n$, with heterogeneous variances across levels of T. While for each t_j there are many realizations of D, the structure $t_j D$ produces piecewise linear segments (Figure S1). Consequently, for a fixed d_k, each observation $t_i d_k$ originates from a different conditional distribution. This structure violates the assumptions of standard asymptotic theory, such as those underlying the Central Limit Theorem in regression contexts, and complicates the interpretation of standard errors.

As a result, error terms in the regression equation cannot be regarded as independent random errors. This indicates that the equation $DT = t_0 D + k$ is best viewed as a numerical trick for obtaining point estimates rather than a probabilistic model. While the method remains algebraically correct and can provide point estimates of t_0 and k, the associated error terms lack a straightforward probabilistic or biological interpretation.

In summary, Ikemoto and Takai's method may still be useful when the goal is to obtain point estimates. However, due to its sensitivity to experimental design and the structural dependence of the variables involved, it does not provide a solid basis for assessing uncertainty.

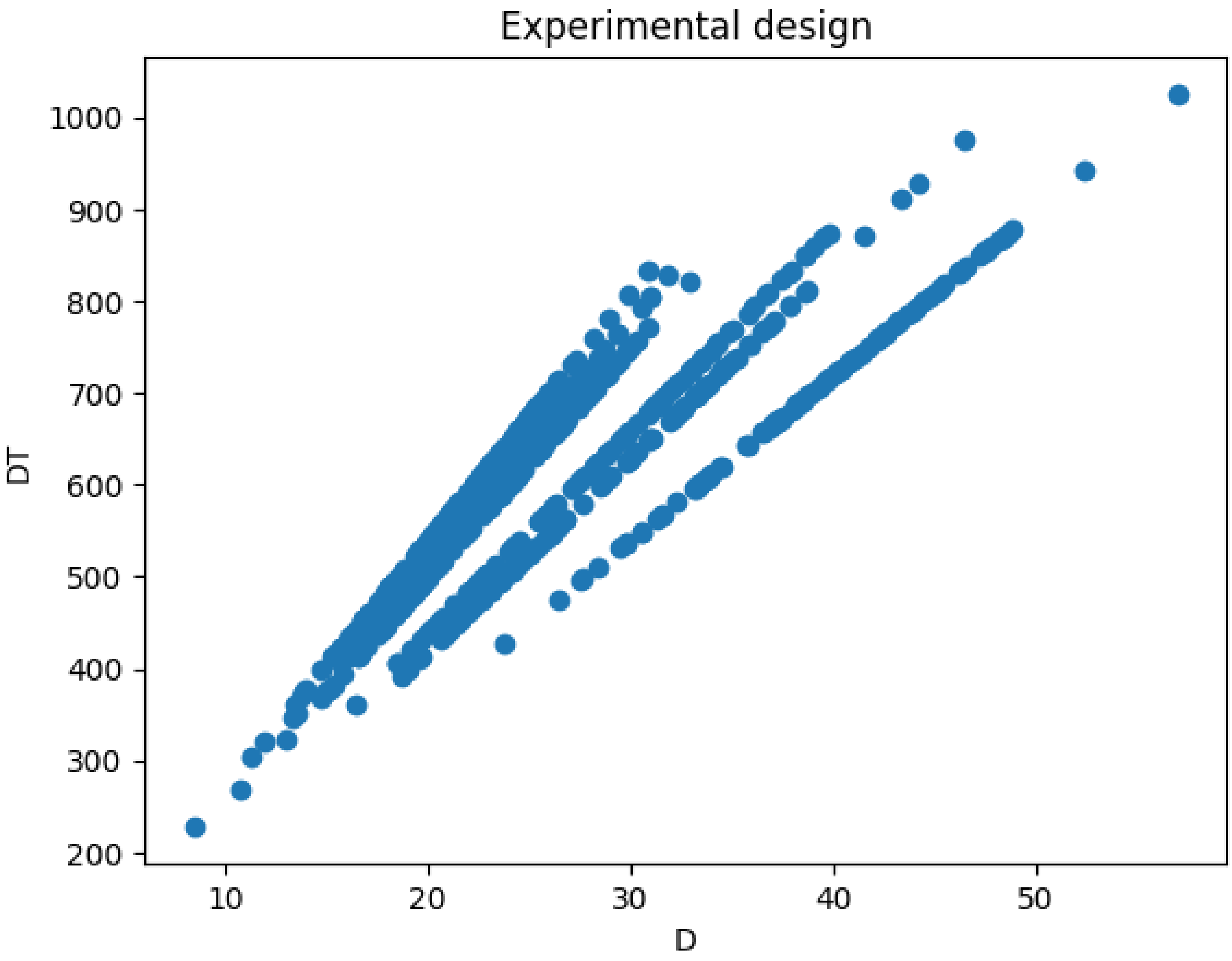

Figure S1. Illustration of the structural dependence between D and DT in simulated developmental data under the Ikemoto and Takai framework

Additional results for *Creophilus maxillosus*

The population of outliers shows a systematically longer time of development (Figure S2).

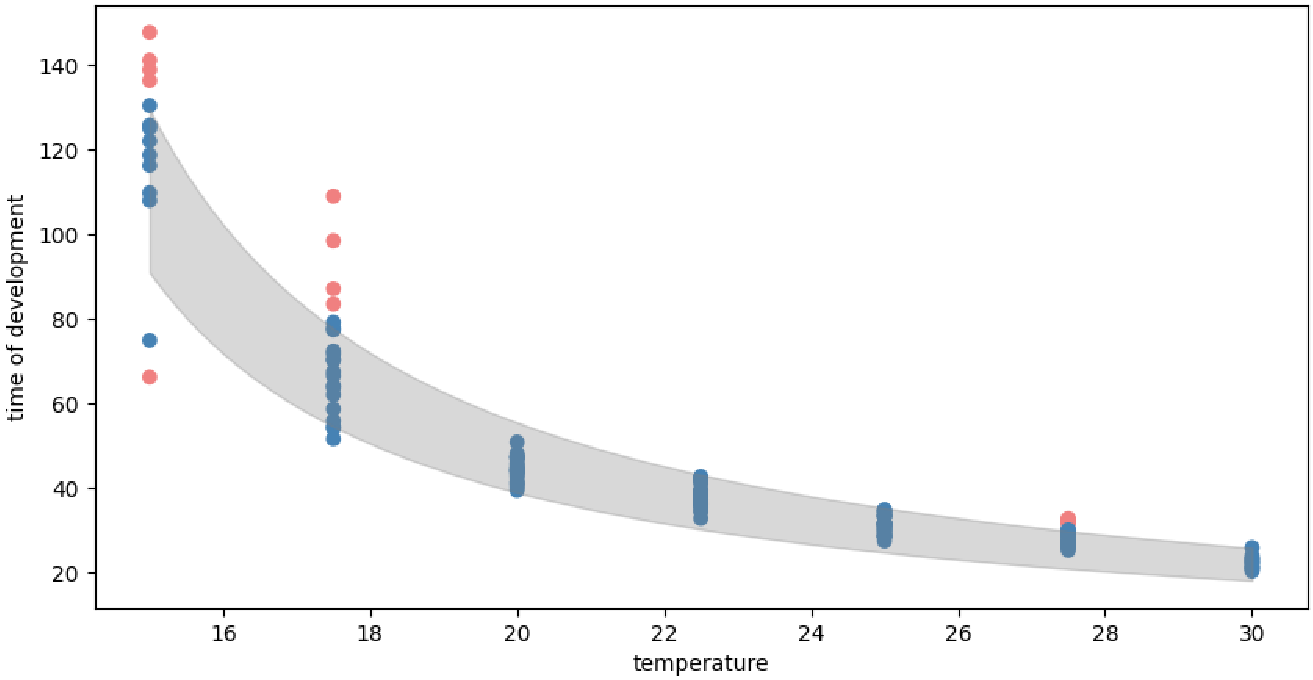

Figure S2. Time of development vs. temperature in the *Creophilus maxillosus*' dataset. Blue points are regular results of the experiment, red points are outliers, and the shaded area is an interval estimate.

Confidence interval

The 95% confidence interval for the parameter k estimated using the Ikemoto and Takai's method is $[378, 456]$, while its empirical counterpart derived from the bootstrap sample is $[405, 423]$. The empirical coverage probability of this 95% confidence interval is 1. The standard error of the Ikemoto and Takai's estimator is 19.52, while the bootstrap standard error is 4.96 (Table S1).

For the new method, the 95% confidence interval of the estimator for the expected value of K (the counterpart of the parameter k from the Ikemoto and Takai's method) is $[416.641, 428.709]$, while its empirical counterpart derived from the bootstrap sample is $[416.972, 428.709]$. The empirical coverage probability of this 95% confidence interval is 0.96. The standard error of the new estimator is 3.02, whereas the bootstrap equivalent is 3.06 (Table S1).

Table S1. Comparison of confidence intervals for *Creophilus maxillosus* derived using different methods. The table presents the interval bounds, their width, and the empirical coverage probability.

Method	95% confidence interval	Interval width	Coverage probability (95% interval)
Ikemoto and Takai	[378, 456]	78.00	1.00
This paper	[416.641, 428.709]	12.07	0.96

Additional calculations of insect age estimation

The comparison of the performance between the proposed method and the Ikemoto and Takai's method in estimating the insect age across different temperatures for the combined population of *Creophilus maxillosus* is presented in Table S2. Additionally, Table S3 shows the performance of the new method applied exclusively to the regular-insect population (blue), while Table S4 presents the results for the outlier population (red).

Table S2. Comparison of coverage probabilities for the proposed method (using combined population) and the Ikemoto and Takai's method at various temperatures for *Creophilus maxillosus*.

Temperature [°C]	Proposed method	Ikemoto and Takai method
15.0	0.86	0.43
17.5	0.81	0.33
20.0	1.00	0.38
22.5	1.00	0.70
25.0	1.00	0.83
27.5	1.00	0.56
30.0	1.00	0.85

Table S3. Coverage probabilities of the proposed method for the regular-insect population (blue) of *Creophilus maxillosus*.

Temperature [°C]	Proposed method (regular-insect population)
15.0	0.78
17.5	0.71
20.0	1.00
22.5	1.00
25.0	1.00
27.5	0.91
30.0	0.92

Table S4. Coverage probabilities of the proposed method for the outlier population (red) of *Creophilus maxillosus*.

Temperature [°C]	Proposed method (outlier population)
15.0	0.80
17.5	0.75
27.5	1.00

Additional results of *Necrodes littoralis*

Population of small insects shows a systematically longer time of development (Figure S3).

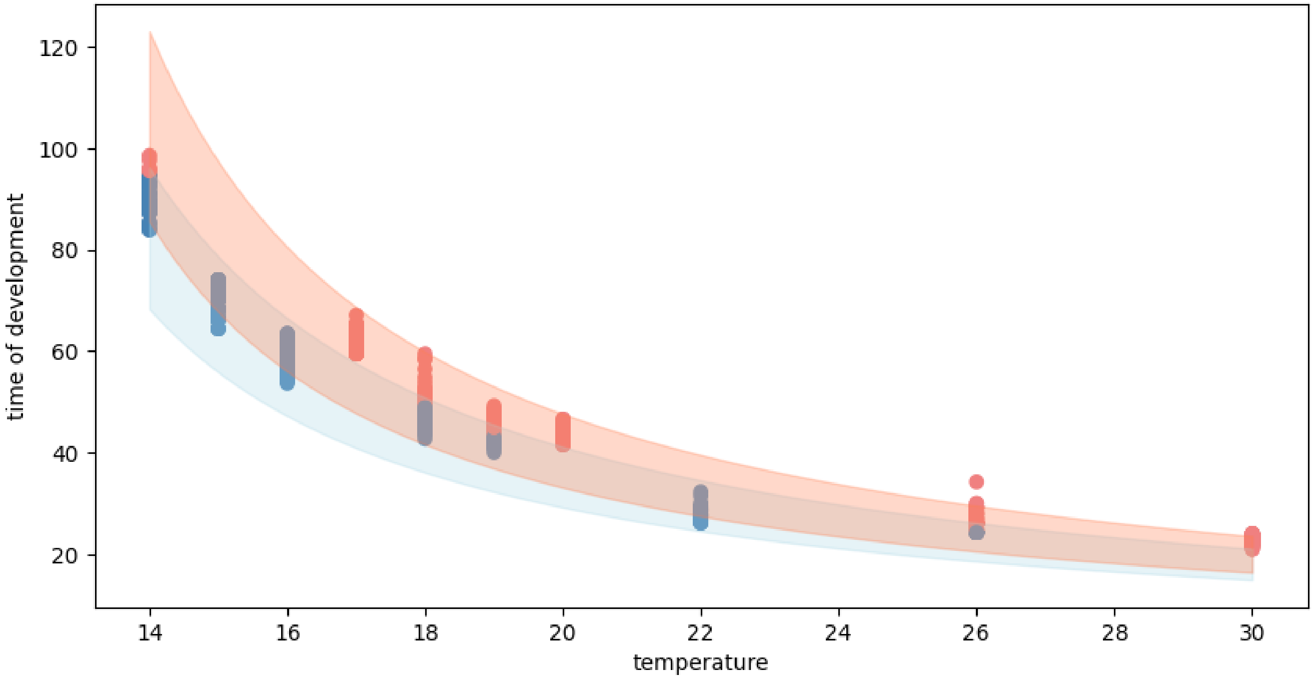

Figure S3. Time of development vs. temperature in the *Necrodes littoralis*' dataset. Blue points represent the population of large insects, red points represent the population of small insects, and the shaded area indicates an interval estimate.

Confidence interval

The 95% confidence interval for the parameter k estimated using the Ikemoto and Takai's method is $[420, 518]$, while its empirical counterpart derived from the bootstrap sample is $[464, 468]$. The empirical coverage probability of this 95% confidence interval is 1. The standard error of the Ikemoto and Takai's estimator is 24.59, while the bootstrap standard error is 0.9.

For the new method, the 95% confidence interval of the estimator for the expected value of K (the counterpart of the k parameter from Ikemoto and Takai's method) for the large-insect population is $[376.786, 381.031]$, while its empirical counterpart derived from the bootstrap sample is $[376.874, 381.147]$. The empirical coverage probability of this 95% confidence interval is 0.95. The standard error of the new estimator is 1.06, whereas the bootstrap equivalent is 1.05. Next, for the small-insect population, the interval is $[422.713, 426.951]$, while its empirical counterpart derived from the bootstrap sample is $[422.645, 427.058]$. The empirical coverage probability of this 95% confidence interval is 0.94. The standard error of the new estimator is 1.06, whereas the bootstrap equivalent is 1.09. Finally, for the combined population, the interval is $[400.336, 404.560]$, while its empirical counterpart derived from the bootstrap sample is $[400.475, 404.645]$. The empirical coverage probability of this 95% confidence interval is 0.95. The standard error of the new estimator is 1.06, whereas the bootstrap equivalent is 1.08 (Table S5).

Additional calculations of insect age estimation

The comparison of the performance between the proposed mixture-model-based method and the Ikemoto and Takai's method in estimating the insect age across different temperatures for the combined population of *Necrodes littoralis* is presented in Table S6. Additionally, Table S7 shows the performance of the new method applied exclusively to the large-insect population (blue), while Table S8 presents the results for the small-insect population (red).

Table S5. Comparison of confidence intervals for *Necrodes littoralis* derived using different methods. The table presents the interval bounds, their width, and the empirical coverage probability.

Method	95% confidence interval	Interval width	Coverage probability (95% interval)
Ikemoto and Takai	[420, 518]	98.00	1.00
This paper (large insects, blue population)	[376.786, 381.031]	4.25	0.95
This paper (small insects, red population)	[422.713, 426.951]	4.24	0.94

Table S6. Comparison of coverage probabilities for the proposed method (using combined population) and the Ikemoto and Takai's method at various temperatures for *Necrodes littoralis*.

Temperature [°C]	Proposed method	Ikemoto and Takai method
14.0	1.00	0.87
15.0	1.00	0.96
16.0	1.00	0.77
17.0	1.00	0.26
18.0	0.99	0.91
19.0	1.00	0.99
20.0	1.00	0.79
22.0	1.00	0.02
26.0	0.97	0.97
30.0	0.79	1.00

Table S7. Coverage probabilities of the proposed method for the large-insect population (blue) of *Necrodes littoralis*.

Temperature [°C]	Proposed method (large-insect population)
14.0	1.00
15.0	1.00
16.0	1.00
18.0	1.00
19.0	1.00
22.0	1.00
26.0	1.00

Table S8. Coverage probabilities of the proposed method for the small-insect population (red) of *Necrodes littoralis*

Temperature [°C]	Proposed method (small-insect population)
14.0	1.00
17.0	1.00
18.0	1.00
19.0	1.00
20.0	1.00
26.0	0.96
30.0	0.82

Additional discussion about construction of confidence intervals

The Ikemoto and Takai's method[1] appears to perform poorly in the construction of confidence intervals. The intervals it produces are excessively wide and diverge substantially from those obtained via bootstrap sampling[2], a standard approach when the underlying sampling distribution is unknown or analytically intractable. For this reason, we adopted bootstrap-based intervals as a reference.

The inadequacy of the Ikemoto and Takai method's error estimation was particularly evident in the case of *Necrodes littoralis*. Here, the bootstrap procedure yielded a very small standard error, indicating substantial potential for precise parameter estimation; precision that is not reflected in the standard error formula used in the original method. Additional results illustrating this discrepancy are provided in the Supplementary Materials.

The confidence intervals derived from bootstrap sampling[2] and those obtained using the proposed method are very similar, which supports the conclusion that the new method provides high precision. In contrast, the coverage probability of the confidence intervals produced by the Ikemoto and Takai's method for both species examined here is equal to 1, indicating that these intervals are excessively wide. The coverage probability for a 95% interval should be close to 0.95, as is the case with the proposed method. Otherwise, the intervals should be considered inaccurate[3] (for more details, see additional results in the Supplementary Materials).

Example of practical application using *Necrodes littoralis* data

Estimation of time of development

Suppose an expert found a freshly eclosed individual of the species *Necrodes littoralis* on the cadaver, measuring 18 mm in length, and the average preceding temperature was 22 degrees Celsius, hence $T = 22$. Let k_l and k_u denote the lower and upper bounds of the interval estimate of K, respectively. The insect age values calculated using the Ikemoto and Takai method are $k_l = 420$, $k_u = 518$ ($\approx 468.89 \pm 2 \cdot 24.59$), and $t_0 = 8.49$[4]. Ultimately, the interval estimate of the development time is:

$$\left[\frac{k_l}{T-t_0}, \frac{k_l}{T-t_0}\right] = \left[\frac{420}{22-8.49}, \frac{518}{22-8.49}\right] = [31.01, 38.34] \quad (1)$$

In our dataset on *Necrodes littoralis*[5], at a temperature of 22°C, two individuals (approximately 2%) with a length of 18 mm or more developed within the interval [31.01, 38.34] days.

Considering the same scenario but using the values calculated with the method from this paper, namely $k_l = 306$, $k_u = 431$ (Table S12), and $t_0 = 9.52$ (Table S9). In this case, the parameters of the large-insect population (blue) should be used, as the length of the examined individual exceeds the average length of individuals in the large-insect population (blue) (Table S10). The interval estimate of the development time is:

$$\left[\frac{k_l}{T-t_0}, \frac{k_l}{T-t_0}\right] = \left[\frac{306}{19-9.52}, \frac{431}{19-9.52}\right] = [24.52, 34.54] \quad (2)$$

In the dataset, all individuals measuring 18 mm or more at a temperature of 22°C developed within the interval [24.52, 34.54] days.

Controlling width of the interval estimate

According to the assumption adopted in the article, the above intervals should cover approximately 95% of the actual cases. Matuszewski and Mądra-Bielewicz[6] suggest that in some cases, using narrower intervals may be more practical. With the Ikemoto and Takai method, it is not possible to control how many actual cases will be covered by the interval. In contrast, with the new method, intervals covering any desired proportion of cases can be calculated using the quantiles of the Weibull distribution with parameters from Table S9. Tables S11 to S15 contain examples.

Table S9. Coefficients of the Weibull mixture model for *Necrodes littoralis*. Reproduced from the main article (Table 4). The table presents estimated parameters for each component population.

Parameter	Large-insect population (blue)	Small-insect population (red)
w_l	0.41	0.59
κ_l	14.57	13.67
λ_l	394.44	423.95
$t_0^{(l)}$	9.52	10.21

Table S10. Mean insect length in each population of *Necrodes littoralis*. Reproduced from the main article (Table 5).

Population	Length [mm]
Large-insect population (blue)	17.75
Small-insect population (red)	16.96

Table S11. Examples of interval estimates of k for *Necrodes littoralis* individuals smaller than 17.34 mm. The table presents different quantile orders used to define interval bounds, the resulting interval estimates, and their theoretical coverage probabilities.

Quantile orders of the interval limits	Interval estimate	Theoretical coverage probability
[0.025, 0.975]	[324, 466]	0.95
[0.05, 0.95]	[341, 459]	0.90
[0.1, 0.9]	[360, 451]	0.80
[0.15, 0.85]	[371, 444]	0.70

Table S12. Examples of interval estimates of k for *Necrodes littoralis* individuals larger than 17.34 mm. The table presents different quantile orders used to define interval bounds, the resulting interval estimates, and their theoretical coverage probabilities.

Quantile orders of the interval limits	Interval estimate	Theoretical coverage probability
[0.025, 0.975]	[306, 431]	0.95
[0.05, 0.95]	[322, 425]	0.90
[0.1, 0.9]	[338, 418]	0.80
[0.15, 0.85]	[348, 412]	0.70

Table S13. Examples of interval estimates of k for the combined population of *Necrodes littoralis*. The table shows how the quantile orders affect the resulting interval estimates and their theoretical coverage probabilities.

Quantile orders of the interval limits	Interval estimate	Theoretical coverage probability
[0.025, 0.975]	[315, 461]	0.95
[0.05, 0.95]	[331, 453]	0.90
[0.1, 0.9]	[348, 442]	0.80
[0.15, 0.85]	[359, 435]	0.70

Table S14. Examples of interval estimates of k for *Creophilus maxillosus*. The table presents different quantile orders with their corresponding interval estimates and theoretical coverage probabilities.

Quantile orders of the interval limits	Interval estimate	Theoretical coverage probability
[0.025, 0.975]	[341, 486]	0.95
[0.05, 0.95]	[358, 479]	0.90
[0.1, 0.9]	[377, 470]	0.80
[0.15, 0.85]	[389, 463]	0.70

Table S15. Examples of interval estimates of k for *Creophilus maxillosus*, including the outlier population. The table presents different quantile orders with corresponding interval estimates and theoretical coverage probabilities.

Quantile orders of the interval limits	Interval estimate	Theoretical coverage probability
[0.025, 0.975]	[339, 600]	0.95
[0.05, 0.95]	[358, 553]	0.90
[0.1, 0.9]	[378, 489]	0.80
[0.15, 0.85]	[391, 475]	0.70

Technical details

Assumptions

We assumed that (D,T) is a bivariate random variable, where the variables D and T are related according to the formula:

$$K = D(T - t_0), \tag{3}$$

where K is a random variable of a development parameter k, whereas k is a realization of K.

We assumed that combined population K_c consists of component populations $K_1, K_2, \ldots, K_L$. Each K_i is distributed accoring to Weibull distribution (as the Weibull distribution is used for modeling time-to-event data[7] and K is a time, normalized by temperature, to completion of development), and consequently, the combined population follows a finite mixture model[8] of Weibull distributions, i.e.:

$$\begin{aligned} K_1 &\sim \mathrm{Weib}(k_1, \kappa_1, \lambda_1), \\ K_2 &\sim \mathrm{Weib}(k_2, \kappa_2, \lambda_2), \\ &\vdots \\ K_L &\sim \mathrm{Weib}(k_L, \kappa_L, \lambda_L), \\ K_c &\sim \sum_{i=1}^{L} w_i \, \mathrm{Weib}(k_i, \kappa_i, \lambda_i) \end{aligned} \tag{4}$$

where K_i are random variables, k_i are their realizations, weib is the density of the Weibull distribution, κ_i and λ_i are the parameters of the Weibull distribution, $\sum_{i=1}^{L} w_i = 1$.

Moreover, we assume that $k_i = d(t - t_0^{(i)})$, where d is a realization of the random variable D, t is a realization of random variable T, $t_0^{(i)}$ is a parameter that distinguishes populations. In consequence:

$$\begin{aligned} K_1 &= D(T - t_0^{(1)}) \sim c_1 \, \mathrm{Weib}(d(t - t_0^{(1)}), \kappa_1, \lambda_1), \\ K_2 &= D(T - t_0^{(2)}) \sim c_2 \, \mathrm{Weib}(d(t - t_0^{(2)}), \kappa_2, \lambda_2), \\ &\vdots \\ K_L &= D(T - t_0^{(L)}) \sim c_L \, \mathrm{Weib}(d(t - t_0^{(L)}), \kappa_L, \lambda_L), \\ K_c &\sim \sum_{i=1}^{L} w_i \, c_i \, \mathrm{Weib}(d(t - t_0^{(i)}), \kappa_i, \lambda_i) \end{aligned} \tag{5}$$

where c_i are normalization constants, the remaining notations are as previously defined.

Finally, our goal is to estimate the parameters of the distribution of the combined population, i.e. $t_0^{(i)}$, κ_i, and λ_i, for $i = 1, \ldots, L$.

Fitting model

We applied the EM (Expectation–maximization) algorithm[9]. It is iterative, so we randomly chose starting values for the parameters. Let

$$
\begin{aligned}
\mathbf{w} &= (w_1, \ldots, w_L) \\
\boldsymbol{\kappa} &= (\kappa_1, \ldots, \kappa_L) \\
\boldsymbol{\lambda} &= (\lambda_1, \ldots, \lambda_L) \\
\mathbf{t_0} &= \left(t_0^{(1)}, \ldots, t_0^{(L)}\right) \\
{}^{(j-1)}\mathbf{w} &= \left({}^{(j-1)}w_1, \ldots, {}^{(j-1)}w_L\right) \\
{}^{(j-1)}\boldsymbol{\kappa} &= \left({}^{(j-1)}\kappa_1, \ldots, {}^{(j-1)}\kappa_L\right) \\
{}^{(j-1)}\boldsymbol{\lambda} &= \left({}^{(j-1)}\lambda_1, \ldots, {}^{(j-1)}\lambda_L\right) \\
{}^{(j-1)}\mathbf{t_0} &= \left({}^{(j-1)}t_0^{(1)}, \ldots, {}^{(j-1)}t_0^{(L)}\right) \\
\mathbf{d} &= (d_1, \ldots, d_n) \\
\mathbf{t} &= (t_1, \ldots, t_n)
\end{aligned}
\tag{6}
$$

The vectors $\mathbf{w}$, $\boldsymbol{\kappa}$, $\boldsymbol{\lambda}$, and $\mathbf{t_0}$ are the vectors of parameters we want to estimate. The vectors $\mathbf{w}^{(j-1)}$, $\boldsymbol{\kappa}^{(j-1)}$, $\boldsymbol{\lambda}^{(j-1)}$, and $\mathbf{t_0}^{(j-1)}$ are the approximations of these parameters in the $(j-1)$-th iteration. The vectors $\mathbf{d}$ and $\mathbf{t}$ represent the data.

The EM algorithm maximizes the Q-function. In our case, it is of the following form:

$$
\begin{gathered}
Q(\mathbf{w}, \boldsymbol{\kappa}, \boldsymbol{\lambda}, \mathbf{t_0} \mid {}^{(j-1)}\mathbf{w}, {}^{(j-1)}\boldsymbol{\kappa}, {}^{(j-1)}\boldsymbol{\lambda}, {}^{(j-1)}\mathbf{t_0}, \mathbf{d}, \mathbf{t}) = \\
\sum_{i=1}^{n} \sum_{l=1}^{L} v_{i,l} \left({}^{(j-1)}\mathbf{w}, {}^{(j-1)}\boldsymbol{\kappa}, {}^{(j-1)}\boldsymbol{\lambda}, {}^{(j-1)}\mathbf{t_0}\right) \left[\log(w_l) + \log\left(c_l \operatorname{Weib}(d_i(t_i - t_0^{(l)}), \kappa_l, \lambda_l)\right)\right]
\end{gathered}
\tag{7}
$$

where

$$
v_{i,r}\left({}^{(j-1)}\mathbf{w}, {}^{(j-1)}\boldsymbol{\kappa}, {}^{(j-1)}\boldsymbol{\lambda}, {}^{(j-1)}\mathbf{t_0}\right) = \frac{{}^{(j-1)}w_r \, c_r \operatorname{Weib}\left(d_i(t_i - {}^{(j-1)}t_0^{(r)}), {}^{(j-1)}\kappa_r, {}^{(j-1)}\lambda_r\right)}{\sum_{l=1}^{L} {}^{(j-1)}w_l \, c_l \operatorname{Weib}\left(d_i(t_i - {}^{(j-1)}t_0^{(l)}), {}^{(j-1)}\kappa_l, {}^{(j-1)}\lambda_l\right)}
\tag{8}
$$

We then manually computed the partial derivatives of the Q-function with respect to each parameter. (This is a minor difference from the approach described by McLachlan et al.[8]. They did not differentiate with respect to the parameter w, but we did). In each iteration, we computed the value of the Q-function and found its stationary point by founding the zeros of the partial derivatives using the bisection method. The stationary point was then used as the parameter values for the next iteration. The loop terminated when the difference in the Q-function values between consecutive iterations was less than 10^{-6}.

We denote the estimated values of the parameters as $\widehat{w}_i$, $\widehat{t}_0^{(i)}$, $\widehat{\kappa}_i$, and $\widehat{\lambda}_i$.

Clustering

To separate the combined population into component populations, we performed a clustering based on the following principle: a point (d_i, t_i) belongs to population l if:

$$
\widehat{w}_l \, c_l \operatorname{Weib}\left(d_i(t_i - \widehat{t}_0^{(l)}), \widehat{\kappa}_l, \widehat{\lambda}_l\right) = \max\left(\widehat{w}_1 \, c_1 \operatorname{Weib}(d_i(t_i - \widehat{t}_0^{(1)}), \widehat{\kappa}_1, \widehat{\lambda}_1), \ldots, \widehat{w}_L \, c_L \operatorname{Weib}(d_i(t_i - \widehat{t}_0^{(L)}), \widehat{\kappa}_L, \widehat{\lambda}_L)\right)
\tag{9}
$$

From now on, we refer to $k_i = d_i(t_i - t_0^{(l)})$ as the ”true k”.

Choosing number of populations

To determine the number of component populations, we applied the EM algorithm four times with L set to 1, 2, 3, and 4, respectively. We then selected the optimal value of L using the Bayesian Information Criterion (BIC) and the elbow rule[10]. The formula for BIC was as follows:

$$\mathrm{BIC}(m,n,f,\mathbf{d},\mathbf{t}) = m\ln(n) - 2\sum_{i=1}^{n}\ln\left(f(d_i,t_i)\right) \tag{10}$$

where m is the number of parameters, n is the number of observations, f is the density of the combined population, and $\mathbf{d}$, $\mathbf{t}$ are vectors of data as previously defined.

Choosing the best population

Our goal in this part is to select one population, which we then use to calculate both the interval estimate and the confidence interval.

If, in a given case, the value w_l for a certain component population was much higher than for the others, we treated the population l as representing the desirable experimental results, and the remaining populations as outlier observations. The meaning of "much higher" is subjective and arbitrary.

If the values w_i for several populations were high and similar, we used a Mann-Whitney U-test to assess the significance of differences in the sizes of individuals belonging to different populations. From further analyses, both the outlier population and the population of small insects were excluded.

Bootstrap sampling

We compared the results of both methods with the results of bootstrap simulations to assess which are more reliable. For the new method, we drew a sample with replacement equal in size to the dataset, transformed the data using the formula $d_i(t_i - t_0^{(l)})$, and then calculated the arithmetic mean. We repeated this sampling 1,000 times, obtaining a bootstrap sample, which we used to determine an empirical confidence interval for the mean. We refer to this confidence interval as the empirical counterpart derived from the bootstrap sample ("counterpart" because we calculate the confidence interval using the proposed method). In the case of the Ikemoto and Takai method, we also drew a sample with replacement equal in size to the dataset and then applied the Ikemoto and Takai method to this new sample. By repeating this sampling 1,000 times, we obtained a bootstrap sample, which we used to determine an empirical confidence interval for the parameter k. We also refer to this confidence interval as the empirical counterpart derived from the bootstrap sample, which should not lead to misunderstandings (again "counterpart" because we calculate the confidence interval using the Ikemoto and Takai's method).

It is easy to show that both approaches lead to a distribution of the mean which, based on the central limit theorem, converges to a normal distribution as the sample size increases. However, these distributions differ because the Ikemoto and Takai method manipulates the sample size, which slows down the convergence.

Coverage probability of confidence interval

To evaluate whether the presented method is more precise than the Ikemoto and Takai[1] method, we calculated the empirical coverage probability for the population l, selected as described earlier.

We computed the confidence intervals for the expected value of the variable $D(T - t_0^{(l)})$ based on the sample $\left(d_1(t_1 - t_0^{(l)}),\ldots,d_n(t_n - t_0^{(l)})\right)$ using the Central Limit Theorem, i.e.:

$$\left[\overline{k} - 1.96\,\frac{\widehat{s}}{\sqrt{n}},\ \overline{k} + 1.96\,\frac{\widehat{s}}{\sqrt{n}}\right] \tag{11}$$

where $\overline{k}$ and $\widehat{s}$ are the sample mean and the sample standard deviation of $\left(d_1(t_1 - t_0^{(l)}), d_2(t_2 - t_0^{(l)}), \ldots, d_n(t_n - t_0^{(l)})\right)$. Then we calculated the empirical coverage probability using bootstrap sampling as the proportion of elements of the bootstrap sample that fall inside this interval relative to the total number of elements.

For the Ikemoto and Takai method, we calculated the confidence intervals for the estimated development parameter k, assuming the 95% interval takes the form $[\widehat{k} - 1.96\,s_e,\ \widehat{k} + 1.96\,s_e]$, where $\widehat{k}$ is the parameter estimate and s_e is the standard error, both computed using the Ikemoto and Takai method. For comparison, as we described earlier, we also calculated the empirical coverage probability using bootstrap sampling.

Coverage probability of insect age estimation

Let the random variable X be distributed according to some probability distribution P. We define the 95% interval estimate for realizations x as the interval $[x_l, x_u]$, where x_l and x_u are the 0.025 and 0.975 quantiles of P, respectively.

To obtain the interval estimate for a given realization k of the random variable K, we computed the 0.025 and 0.975 quantiles of the Weibull mixture distribution, using the model parameters estimated in the present study. This yielded a 95% interval that reflects the uncertainty inherent in the mixture-based approach.

To further assess the performance of our method in interval estimation, we additionally calculated quantiles for each component population separately. These were based on single Weibull distributions fitted to the individual component population, identified using the clustering procedure described earlier.

For comparison purposes, we evaluated the empirical coverage probability of each interval estimate by calculating the proportion of observations falling within the respective interval. For the interval derived from the Weibull mixture model, the full dataset was used. For the intervals based on component populations, we used only the observations assigned to the corresponding clusters.

References

1. Ikemoto, T. & Takai, K. A new linearized formula for the law of total effective temperature and the evaluation of line-fitting methods with both variables subject to error. *Environ. Entomol.* **29**, 671–682, DOI: http://dx.doi.org/10.1603/0046-225X-29.4.671 (2000).
2. Efron, B. & Tibshirani, R. J. *An Introduction to the Bootstrap* (Chapman and Hall/CRC, 1994).
3. Casella, G. & Berger, R. *Statistical Inference* (CRC Press, 2024).
4. Gruszka, J. & Matuszewski, S. Temperature models of development for *Necrodes littoralis* l. (coleoptera: Silphidae), a carrion beetle of forensic importance in the palearctic region. *Sci. Reports* **12**, 9689, DOI: https://doi.org/10.1038/s41598-022-13901-y (2022).
5. Gruszka, J. & Matuszewski, S. Estimation of physiological age at emergence based on traits of the forensically useful adult carrion beetle *Necrodes littoralis* l. (silphidae). *Forensic Sci. Int.* **314**, 110407, DOI: https://doi.org/10.1016/j.forsciint.2020.110407 (2020).
6. Matuszewski, S. & Mądra-Bielewicz, A. Field validation of post-mortem interval estimation based on insect development. part 2: Pre-appearance interval, expert evidence selection and accuracy baseline data. *Forensic Sci. Int.* **367**, 112316, DOI: https://doi.org/10.1016/j.forsciint.2024.112316 (2025).
7. Lai, C. D., Murthy, D. N. & Xie, M. Weibull distributions and their applications. In *Springer Handbooks*, 63–78, DOI: https://doi.org/10.1007/978-1-84628-288-1_3 (Springer, 2006).
8. McLachlan, G. J., Lee, S. X. & Rathnayake, S. I. Finite mixture models. *Annu. Rev. Stat. Its Appl.* **6**, 355–378, DOI: https://dx.doi.org/10.1146/annurev-statistics-031017-100325 (2019).
9. Ng, S. K., Krishnan, T. & McLachlan, G. J. The em algorithm. In *Handbook of Computational Statistics: Concepts and Methods*, 139–172, DOI: http://dx.doi.org/10.1007/978-3-642-21551-3_6 (Springer, 2012).
10. Wit, E., van den Heuvel, E. & Romeijn, J. W. 'all models are wrong...': an introduction to model uncertainty. *Stat. Neerlandica* **66**, 217–236, DOI: http://dx.doi.org/10.1111/j.1467-9574.2012.00530.x (2012).

Author contributions statement

J.W.: Conceptualization, Writing – review & editing, Software, Investigation, Data curation, Formal analysis, Validation, Visualization, Writing – original draft. Ł.S.: Conceptualization, Writing – review & editing, Supervision. S.M.: Conceptualization, Writing – review & editing, Supervision.

Data availability statement

The data analyzed in this study were obtained from previously published works and are not publicly available due to copyright and data sharing restrictions. Access to these data can be requested from the corresponding authors of the respective publications:

1. Frątczak-Łagiewska, K., Grzywacz, A. & Matuszewski, S. Development and validation of forensically useful growth models for central european population of Creophilus maxillosus l. (coleoptera: Staphylinidae). Int. J. Leg. Medicine 134, 1531–1545, (2020), https://doi.org/10.1007/s00414-020-02275-3

2. Gruszka, J. & Matuszewski, S. Temperature models of development for Necrodes littoralis l. (coleoptera: Silphidae), a carrion beetle of forensic importance in the palearctic region. Sci. Reports 12, 9689, (2022), DOI: https://doi.org/10.1038/s41598-022-13901-y

The datasets were used with appropriate permissions solely for the purposes of this analysis. All additional data, materials, and code generated during this study are openly available in the GitHub repository (https://github.com/Jedrzej-Wydra/survival_analysis_em) and in supplementary materials.

Additional information

The authors declare no competing interests.
